# Glioblastoma *In Vitro* Model That Recapitulates the Influence of the Hyaluronan Molecular Weight in Cancer Cell Motility and Permeability of the Blood-Brain Tumor Barrier

**DOI:** 10.1021/acsbiomaterials.5c01740

**Published:** 2025-12-10

**Authors:** Fabiana Andrade, Vânia I. B. Castro, Sara Amorim, Ana R. Araújo, Olga Martinho, Natália Alves, Rui L. Reis, Ricardo A. Pires

**Affiliations:** † 3B’s Research Group, I3Bs − Research Institute on Biomaterials, Biodegradables and Biomimetics, University of Minho, Headquarters of the European Institute of Excellence on Tissue Engineering and Regenerative Medicine, AvePark, Barco 4805-017, Portugal; ‡ ICVS/3B’s−PT Government Associate Laboratory, Braga, Guimarães 4805-017, Portugal

**Keywords:** glioblastoma, blood-brain barrier, blood-brain tumor barrier, *in vitro* model, hyaluronan

## Abstract

We report a glioblastoma (GBM) *in vitro* model that combines an extracellular matrix (ECM)-mimicking hydrogel, hyaluronan (HA), GBM spheroids, and a blood-brain barrier (BBB) component. The model was designed to study the impact of the HA’s chain size (i.e., molecular weight, Mw) on cancer cell migration and on the permeability of the BBB. U-87 spheroids were encapsulated in alginate (Alg) hydrogels previously loaded with HA of different Mw, i.e., 5 kDa, 700 kDa, and 1.5 MDa, mimicking the tumor microenvironment (TME) of GBM. The results indicate that shorter HA molecules (i.e., 5 kDa) enhance the invasion of U-87 cells, as observed by time-lapse microscopy. Moreover, this increased cellular motility is accompanied by overexpression of cortactin by the U-87 cells confirming an increased cancer invasive character. In contrast, U-87 spheroids encapsulated in hydrogels that presented HA of higher Mw, i.e., 700 kDa and 1.5 MDa, presented reduced motility, being consistent with a limited cancer growth. Furthermore, dextran-based permeability measurements showed that the presence of HA of low Mw (i.e., 5 kDa) led to increased permeability of the BBB component, a feature that is characteristic of the blood-brain tumor barrier (BBTB). In summary, the developed 3D *in vitro* GBM model effectively recapitulates key features of the TME, highlighting the impact of the HA size on cancer cell invasion and BBB/BBTB permeability.

## Graphical abstract

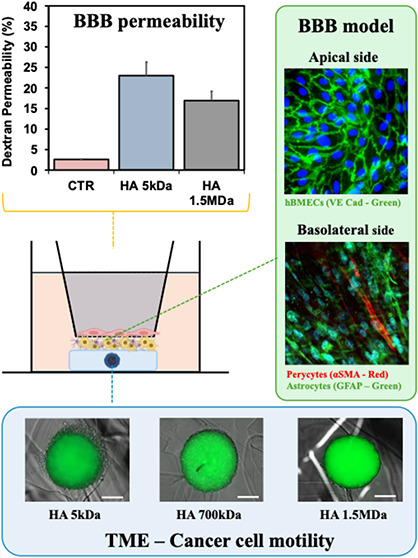

## 1 Introduction

Glioblastoma (GBM) is the most common type of malignant brain tumor in adults, classified as highly aggressive and invasive.^1^ The tumor microenvironment (TME) plays a central role in the development and progression of the disease.^2^ In particular, the brain’s extracellular matrix (ECM) in the vicinity of the GBM displays a large spatial and temporal heterogeneity, with minimal fibrillary structures and a high concentration of hyaluronan (HA).^3^ HA is referred to as one of the main promoters of proliferation and invasion of GBM cancer cells, as well as of cancer resistance to conventional therapies.^4^ Moreover, an impairment in the balance between the synthesis and degradation of HA leads to its accumulation in the TME under various molecular weights (Mw). HA binds to its main cell surface receptors, such as CD44,^5^ activating distinct signaling pathways that are dependent on the HA Mw. While low Mw HA (i.e., <100 kDa) is pro-inflammatory and promotes cancer cells invasion and metastasis, the high Mw HA (i.e., >1000 kDa) induces cancer cell latency.^6,7^

In this context, it is expected that HA of low Mw not only promotes cancer cell invasion and cancer growth, but also impacts the stability of the blood-brain barrier (BBB).^8^ This barrier is formed by a monolayer of endothelial cells in the cerebral capillaries, supported by secondary layers of astrocytes, pericytes, and microglia that, together, are responsible for maintaining the BBB stability and function.^9^ Dissemination of the GBM cells in the vicinity of the BBB triggers the invasion-metastasis cascade mediated by an epithelial to mesenchymal transition (a process by which epithelial cells develop the ability to invade, resist stress, and disseminate).^10^ Consequently, GBM growth triggers the formation of a unique inflammatory and highly vascularized niche of tumor cells near the endothelial cell layer (that composes the BBB) perturbing its stability and impairing the tight junctions (TJs) between the endothelial cells that are responsible for the low permeability of the BBB.^11^ These cascade of events compromise the integrity of the BBB, inducing an increase in its permeability forming the so-called blood-brain-tumor barrier (BBTB).^12^ This BTBB is characterized by a reduction in the expression of TJs, an altered pericyte layer, increased number of reactive astrocytes (characterized by a shrinkage of astrocyte’s endfeet), and a subsequent breakdown of the membrane.^13^ Reports show that HA can mediate this process and contribute to the disruption of the BBB through a CD44-dependent pathway.^14^

3D *in vitro* models have been widely used to mimic the TME, providing deeper insights into the relationship between the TME and cancer progression. In the case of GBM, the inclusion of HA in these models is highly relevant due to the fact that, as previously stated, it mediates cancer cell invasion, formation of the BBTB, among other physiological and pathological processes in the brain.^15^

Herein, we developed a 3D *in vitro* GBM model consisting of U-87 spheroids encapsulated in HA-loaded Alg hydrogels and a BBB component. This platform replicates critical features of the GBM's TME, in particular: the U-87 spheroid copycat the cancer site; while the HA-loaded hydrogel mimics the cancer ECM, where different HA Mw are expected to modulate cancer cell behavior, such as migration or reduced motility. Moreover, the inclusion of the BBB component enables the assessment of the impact of the TME on the formation of the BBTB. Of note, the *in vitro* replication of key features of the BBTB are being pursued by the scientific community in an attempt to have 3D models that better replicate the *in vivo* GBM scenario.

## 2 Results and Discussion

Herein, we developed a GBM *in vitro* model composed of an ECM-mimicking hydrogel loaded with GBM spheroids and studied the impact of the presence of HA (of different Mw) in cancer cell migration and in the permeability of the BBB/BBTB (using as a transwell model).

### 2.1 Preparation and Characterization of the ECM-Mimicking Hydrogels

Initial studies were conducted using alginate (Alg) hydrogels (prepared at a concentration of 20 mg/mL) to which HA of distinct Mw, i.e., 5 kDa, 700 kDa, and 1.5 MDa, were loaded at two different concentrations, i.e., 1 and 2 mg/mL. The selection of the HA Mw was based on the fact that low Mw HA (i.e., <100 kDa) has been associated with increased tumor growth, cell migration, and angiogenesis, whereas high Mw HA (i.e., >1000 kDa) is reported to improve the structural stability of the ECM, reduce tumor growth and angiogenesis, as well as inhibit cancer cell migration.^3^

SEM images of the hydrogels did not show significant structural differences independent of the loading of the HA of different Mw at a concentration of 2 mg/mL (Figure S1). Afterward, rheological assessment of the hydrogels showed that when HA was loaded at a concentration of 1 mg/mL its Mw induced changes in the hydrogel’s stiffness, where an increase of the HA Mw led to an increase in the *G*′ (storage modulus, Figure 1), from approx. 14 kPa (Alg loaded with HA of 5 kDa) to approx. 33 kPa (Alg loaded with HA of 1.5 MDa). This correlation might be related to the ability of the longer HA chains to contribute to the structural integrity of the hydrogel leading to a stiffer material. However, when HA at a concentration of 2 mg/mL was used, no statistically significant differences in the *G*′ and in *G*″ (loss modulus) was observed as a function of the Mw of HA: in all the cases, the *G*′ was between 20 and 23 kPa (Figure 1) and *G*″ was always at approx. 3 kPa. A possible explanation for these observations is that the longer HA chains (i.e., in the Mw of 700 kDa and 1.5 MDa) interfere with the ionic cross-linking of the Alg,^16^ leading to a reduction of stiffness that compensates for the initial increase in stability observed when 1 mg/mL of HA was loaded in the hydrogels.

**Figure 1:**
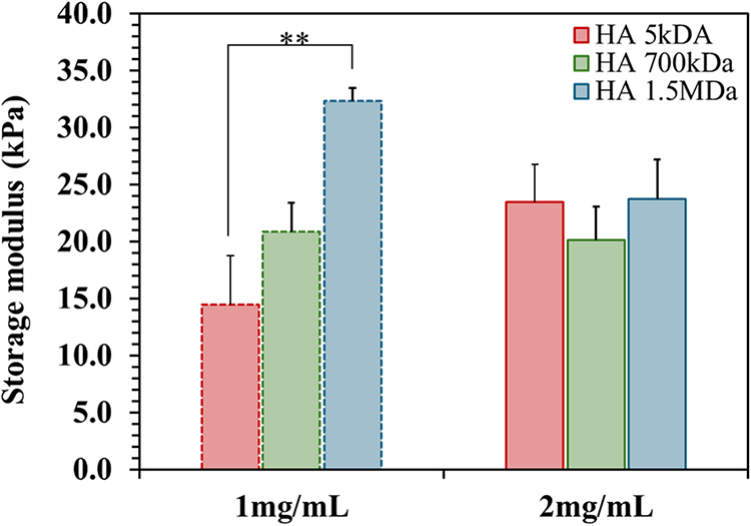
Storage modulus (*G*′) of Alg hydrogels loaded with HA of 5 kDa, 700 kDa, and 1.5 MDa, at HA concentrations of 1 and 2 mg/mL. Results from at least 5 replicas. Statistically significant differences: **p < 0.05.

As the loading of different Mw of HA, at a concentration of 1 mg/mL, alters the mechanical properties of the hydrogels, it would be difficult to separate the biochemical influence of the HA Mw on the cancer cells’ behavior from alterations in mechano-transduction events triggered by differences in the hydrogel stiffness. Moreover, the hydrogel stiffness is also significantly higher than the brain ECM; however, attempts to reduce the concentration of Alg to reduce the stiffness led to similar variations in stiffness as a function of the HA Mw. In this context, we decided to proceed with our studies by maintaining the Alg concentration and load them with only 2 mg/mL of HA to eliminate stiffness variations from our *in vitro* model.

### 2.2 Cancer Cell Migration as a Function of the HA Molecular Weight

*In vivo*, the ECM plays a crucial role in regulating the proliferation, migration, and invasion of cancer cells. As previously mentioned, this is achieved through mechano-transduction events and/or the participation of the ECM components in the biochemical cascades that regulate cellular behavior. HA is a main component of the brain’s ECM and is involved in various phases of tumor growth.^3,17^ Importantly, HA promotes cancer cell migration when present at low Mw, while high Mw HA reduces cancer growth in, for example, gastric cancer.^16^ To assess if our ECM-mimicking hydrogels could recapitulate the ability of the HA Mw to modulate cancer cell behavior in GBM, we loaded U-87 spheroids in the Alg hydrogels that were previously prepared in the presence of HA of the three different Mw, i.e., 5 kDa, 700 kDa, and 1.5 MDa.

GFP-labeled U-87 cells were cultured in an ultralow attachment 96-well plates for the formation of U-87 spheroids. After 3 days of culture, the spheroids were collected and encapsulated in the HA-loaded Alg hydrogels. The migration behavior of the U-87 cancer cells was monitored under time-lapse microscopy using both bright-field and fluorescence imaging during the first 24h after the encapsulation. The collected images allowed us to determine the cell invasion area (Figure 2) by calculating the area of the spheroid projection including the invasive cells of higher motility, while considering the area of the compact spheroid projection edge as a reference (as detailed in Figure S2). Interestingly, the U-87 cells presented a significantly enhanced migration in the presence of HA of 5 kDa, that increased over time, i.e., from 12h to 24h after spheroid’s encapsulation. At 24h, the hydrogels with 5 kDa of HA induces an increase of cell invasion area of, approx. 18%. In contrast, no significant differences were observed between the cell invasion areas determined for the hydrogels presenting HA of higher Mw (i.e., 700 kDa and 1.5 MDa), both remaining close and below, approx. 5%. Importantly, our hydrogel formulations were able to mimic the ability of the HA of different Mw to induce cancer cell invasion (promoted by HA of low Mw) or reduction of cellular motility (promoted by HA of high Mw), recapitulating the *in vivo* scenario as previously shown in different GBM studies.^3,17,18^

**Figure 2:**
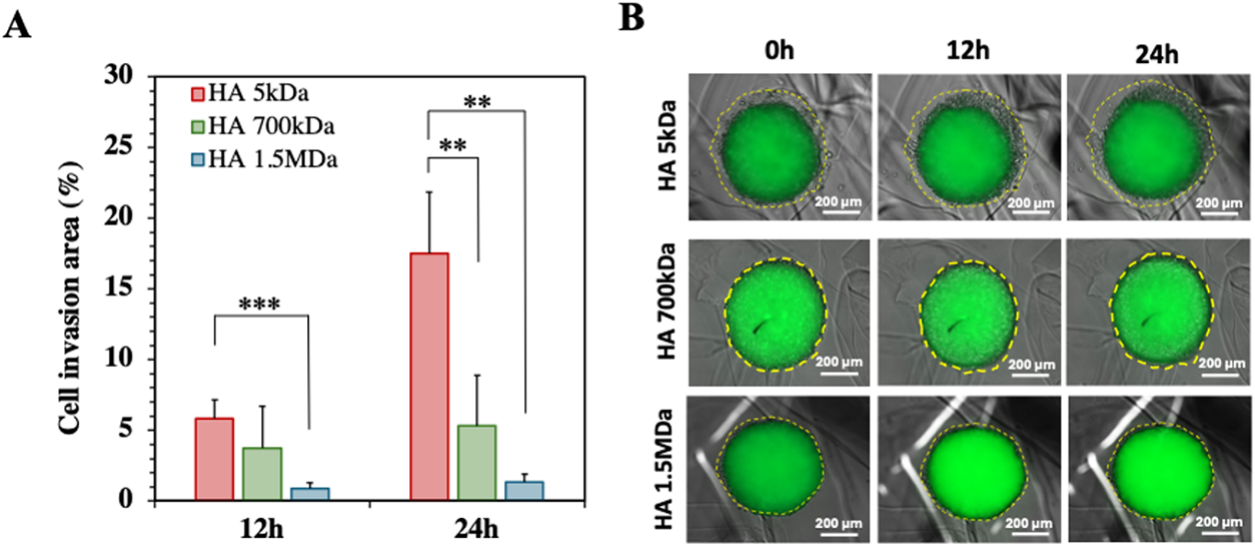
Migration assay of cancer cells from U-87 spheroids encapsulated in the Alg hydrogels loaded with HA of different Mw. **(A**) Quantitative analysis of the percentage of cell invasion after 12 and 24 h of cell culture. (**B)** Representative optical/fluorescence microscopy images of the U-87 spheroids in the presence of HA of different Mw at 0, 12, and 24 h after their encapsulation. Yellow lines indicate the cell invasion area. Scale bar: 200 μm. Results from at least 5 replicas. Statistically significant differences: **p < 0.05 and ***p < 0.01.

### 2.3 Biochemical Characterization of the Cancer Cell Invasive Character

To complement the results from the cell invasion area, we performed immunostaining for the expression of key markers critical for cancer cell migration and adhesion. The expression of CD44 and cortactin in U-87 cells plays an important role in their invasive behavior. CD44 is a transmembrane glycoprotein and one of the main cell surface receptors for HA, playing a crucial role in mediating cell-ECM interactions, tumor progression, and the maintenance of cancer stem cells. It is particularly important in GBM, as it is associated with increased invasiveness and resistance, making it a valuable marker to assess how the HA Mw impacts the cell behavior.^19,20^ However, CD44 on its own cannot be used to assess cancer progression, as it responds to the presence of HA in the pericellular space (that we add in our experimental setup), making it difficult to derive conclusions only by CD44 expression.^21^ On the other hand, cortactin is a protein from the cytoskeleton involved in the formation of actin filaments and cell motility.^22,23^ It is a protein involved in the formation of invadopodia, which is one of the main structures that supports cancer cell migration through the ECM.^24^ While both markers can be used to analyze how the different Mw of HA affects U-87 cell motility and invasive character, we focus our assessment more on the cortactin expression.

To quantify the expression of CD44 and cortactin, we performed Western blots (WB, Figure 3A,B) that showed significantly higher expression of both cortactin and CD44 in the spheroids presenting HA of low Mw (i.e., 5 kDa), when compared to those encapsulated in hydrogels with HA of higher Mw, i.e., 700 kDa and 1.5 MDa. These results are consistent with the promotion of cell invasive character, as well as the induction of the mesenchymal character in cancer cells by the HA of low Mw, which is in line with the measurements of cell invasion area, where the same low Mw HA promoted higher cancer cell motility.

**Figure 3:**
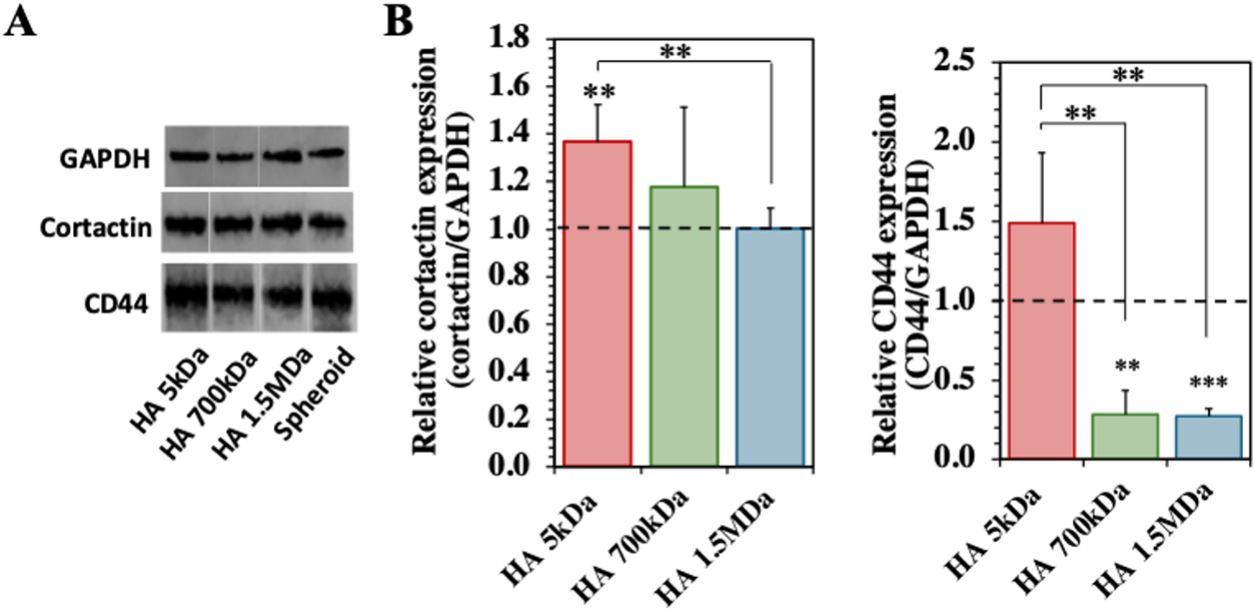
Expression of cortactin and CD44 by U-87 spheroids encapsulated in HA-loaded hydrogels. **(A)** Protein expression quantification by Western-Blotting (WB) of GAPDH (≈35 kDa), cortactin (≈75 kDa), and CD44 (≈81 kDa), at 3 days of cell culture. **(B)** Relative expression of proteins, GAPDH was used as an internal reference control. The statistical differences between HA-loaded hydrogels and spheroid (control) are indicated at the top of each bar, while the statistical difference between different molecular weights of HA (Mw HA) is represented by the line. Results from at least 5 replicas. Statistically significant differences: **p < 0.05 and ***p < 0.01.

In contrast, the spheroids encapsulated in the hydrogels that present HA of higher Mw, i.e., 700 kDa and 1.5 MDa, showed a reduced expression of cortactin (and CD44), suggesting a reduction of the mesenchymal character of the cancer cells and a reduction of cellular motility. These variations in cellular response across the different Mw of HA highlight the crucial role of the HA in regulating GBM cell invasion. Importantly, the developed hydrogels recapitulate these roles, promoting cancer invasion or reduced motility, depending on the Mw of HA being high or low, respectively.

### 2.4 Incorporation of the BBB in the GBM In Vitro Model

The BBB is a highly selective, protective interface formed by endothelial cells, perivascular cells (pericytes), and astrocytes that regulates the passage of nutrients and metabolic byproducts between the blood and the brain. During the progression of GBM, its invasive nature affects the BBB, compromising its integrity and resulting in the formation of the blood-brain tumor barrier (BBTB). The BBTB is characterized by a permeability higher than that of the BBB, which allows an increased flux of nutrients that feeds the cancer site and promotes GBM progression. However, the BBTB, despite being a BBB with higher permeability, still poses significant challenges for the delivery of drugs to the cancer site.^25,26^ Importantly, the invasive behavior of GBM cells plays an important role in the formation of the BBTB by compromising endothelial TJs, leading to the reported increase in permeability.

To recapitulate this process *in vitro*, we increased the complexity of our model and added a BBB layer using a transwell system and coculturing the three main cell types present in the BBB and BBTB, i.e., endothelial cells, pericytes, and astrocytes. Under this setup, the pericytes and astrocytes were cultured on the external layer of the transwell membrane, while in the inner layer we cultured human brain microvascular endothelial cells (hBMECs), known to form a tight monolayer that mimics the BBB. The TJs between these hBMECs are the main factors responsible for the low permeability of the BBB. In GBM, these TJs are compromised leading to the previously mentioned increase of permeability in the BBTB.

To validate this transwell BBB model, we performed immunostaining with specific markers for each cell type. Fluorescence confocal microscopy images confirmed the presence of distinct layers depending on the cell type (Figure S4C). A top layer of hBMECs (apical side, stained for VE-Cadherin in green) is observed, as well as a bottom layer composed of a mixture of pericytes and astrocytes (basolateral side). Astrocytes were immunostained for glial fibrillary acidic protein (GFAP, green), while an antibody against α-smooth muscle actin (αSMA, red) was used to identify the presence of pericytes.

After the establishment of this BBB model, we assessed its integrity by measuring the transendothelial electrical resistance (TEER). Increments in the permeability of the BBB are usually accompanied by a decrease in TEER due to the presence of compromised TJs. After 4 days of culture of the BBB model, the TEER stabilized with values of, approx. 30 Ω^.^cm^2^ (Figure 4A).

**Figure 4:**
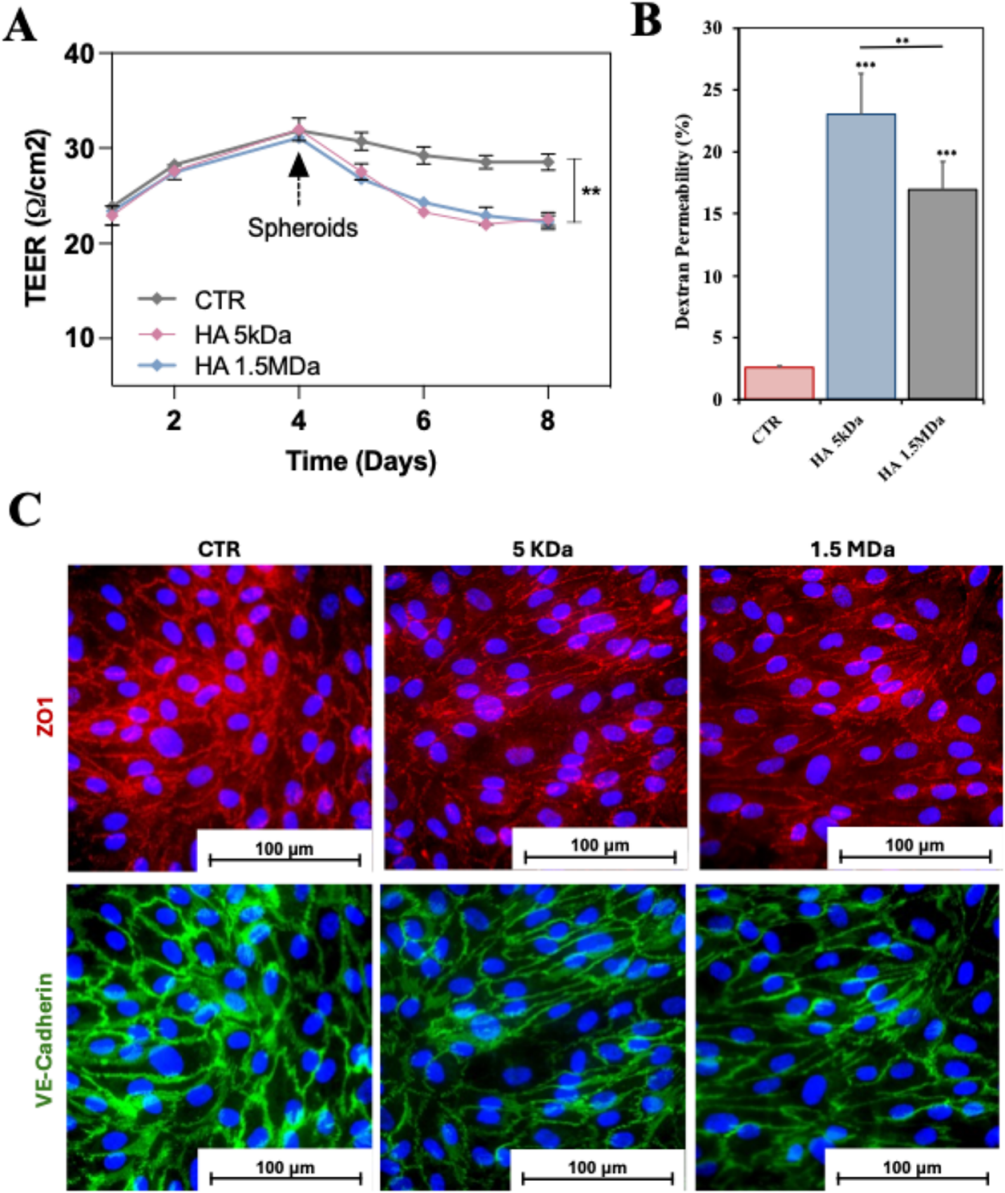
Assessment of the structural integrity of the developed *in vitro* BBB model. (A) Quantification of the TEER as a function of the presence of U-87 spheroids encapsulated in Alg hydrogels that present HA of 5 kDa or HA of 1.5 MDa (control experiment, i.e., CTR, refers to the BBB model in the absence of hydrogel + spheroids). (B) Permeability of the BBB/BBTB model to dextran (Mw of 4 kDa) in the presence of U-87 spheroids encapsulated in HA-loaded hydrogels. (C) Immunostaining of the hBMECs layer for the TJs markers, i.e., ZO-1 and VE-Cadherin, as a function of the HA Mw (control sample corresponds to the BBB model in the absence of spheroid + hydrogel). In (A) and (B) the results are from at least 5 replicas. Statistically significant differences: **p < 0.05 and ***p < 0.01.

Upon confirmation that the selected BBB model generates a barrier of reduced permeability reflected in an increased TEER over time, we combined the BBB model with the ECM-GBM model by placing the HA containing Alg hydrogels (with encapsulated U-87 spheroids) in close proximity to the BBB transwell setup. Of note, the culture media used to prepare the BBB model and to generate the spheroids were different. To guarantee that the spheroids were not affected by the changes in the culture media, we performed the LDH assay on the spheroids cultured in the different media, and no significant cell damage was observed (Figure S3). Moreover, given that our previous results showed that the U-87 spheroids presented a higher invasive character when hydrogels containing HA of 5 kDa were used, and the fact that the hydrogels presenting HA of 1.5 MDa inhibited cancer cell motility, we selected these two experimental conditions to be tested under this setup. Importantly, we checked the HA distribution in the hydrogels, as this is key for the biological assessment studies. We used FITC labeled HA and monitored its presence in the hydrogels by fluorescence microscopy. We found that HA was present and well distributed in the hydrogels over 7 days (Figure S5) in the presence of cell culture media and conditions.

Afterward, hydrogels (presenting HA of 5 kDa or HA of 1.5 MDa) with encapsulated U-87 spheroids were placed at the bottom of the well plate, and the transwell membrane containing the BBB model was added to the top of the hydrogels (avoiding contact to prevent damaging the cell layers), and the TEER was assessed over time, i.e., an additional 4 days (Figure 4A).

TEER values were monitored after exposing the *in vitro* BBB model to the hydrogels combined with U-87 spheroids under different conditions over 8 days (Figure 4A). When the hydrogels with the encapsulated spheroids were added, a significant decrease in TEER over time was observed, to approx. 23 Ω^.^cm^2^. However, no significant differences were detected between the experiments that included HA of 5 kDa and 1.5 MDa. These experiments were complemented by a dextran permeability assay. We quantified the percentage of dextran that crossed the BBB/BBTB model as a function of the presence of spheroids/hydrogel presenting HA of different Mw. This dextran-based assay (Figure 4B) confirmed the higher permeability when the BBB/BBTB was cultured in the spheroids/hydrogel when compared with the control. Moreover, in the case of the hydrogels presenting HA of 5 kDa, this increased permeability is significantly higher than the one detected for the hydrogels that were loaded with HA of 1.5 MDa. A possible explanation for these observations is that the presence of the spheroids+hydrogels loaded with HA of different Mw alters the expression of TJs, as for example, VE-Cadherin and ZO-1. In order to assess if this was in fact, the reason for these observations we performed immunostaining of the hBMECs monolayer to both TJs markers (Figure 4C). While no major changes are observed for the expression of ZO-1, the intensity of the expression of VE-Cadherin is reduced in the presence of the spheroids + hydrogels presenting HA of both Mw. These results are consistent with the TEER and the dextran permeability assessments that showed a compromised endothelial layer that is compatible with a reduction of its stability. Importantly, given the fact that the hydrogels are not in direct contact with the hBMECs monolayer, a possible explanation for our results is the secretion of paracrine factors by the GBM spheroids that affect the stability of the endothelial layer.

## 3 Conclusions

Herein, we designed a 3D GBM *in vitro* model that combines the three main components of the GBM, namely: (1) a HA-loaded Alg-based hydrogel that mimics the brain ECM and that can recapitulate the influence of HA Mw on cancer cell invasion; (2) the cancer site by the encapsulation of U-87 spheroids in the hydrogels; and (3) a BBB component that combines its three main cell types, i.e., hBMECs, astrocytes, and pericytes. We observed that cell invasion and migration were significantly higher in hydrogels that presented HA of lower Mw, i.e., 5 kDa, while the presence of HA of higher Mw (i.e., 700 kDa and 1.5 MDa) restricted cancer cell motility. These observations were confirmed by the overexpression of cortactin, a marker related to cancer cell invasion and invadopodia formation.

Moreover, we also assessed the impact of HA Mw in the BBB-mimicking layers and observed that the presence of the HA-loaded hydrogels and U-87 spheroids led to a reduction in TEER, which is related to increased permeability. This was confirmed by a dextran permeability test showing that all the hydrogels + spheroids increased the BBB permeability, more evident when HA of 5 kDa was used. Moreover, the reduced stability of the BBB (hBMECs monolayer) was also confirmed by a reduced expression of VE-Cadherin in the presence of hydrogels + spheroids. Our data shows that the proposed 3D *in vitro* GBM model (in particular the one presenting HA of low Mw, i.e., 5 kDa) successfully recapitulates key features of GBM, being a valuable platform to study its hallmarks.

## 4 Materials and Methods

### 4.1 Materials and Reagents

Unless otherwise specified, all reagents and solvents were used as received without any additional processing. Hyaluronan (HA) was obtained from Lifecore Biomedical as Sodium hyaluronate with the following molecular weights (Mw): low Mw HA (Sodium hyaluronate with an average Mw of 4800 Da, ref: HA5K-5); medium Mw HA (sodium hyaluronate with an average Mw of 741 kDa, ref: HA700 K-5); high Mw HA (sodium hyaluronate with an average Mw between 1.20 and 1.80 MDa, ref: HA15M-5). Sodium alginate (Alg) was obtained from Pronova (ref: 4200506).

### 4.2 Preparation of Alg-HA-Based Hydrogels (Alg-HA)

To generate the hydrogels, 200 mg of Alg was dissolved in Milli-Q water, to which a solution of HA (of different Mws: 5 kDa, 700 kDa, and 1.5 MDa) in 0.15 M NaCl was added, generating hydrogels with final concentrations of 20 mg/mL of Alg and 1 or 2 mg/mL of HA. The following HA-loadings were assessed: 1 mg/mL 5 kDa-HA; 1 mg/mL 700 kDa-HA; 1 mg/mL 1.5 MDa-HA; 2 mg/mL 5 kDa-HA; 2 mg/mL 700 kDa-HA; and 2 mg/mL 1.5 MDa-HA.

The distribution of HA within the Alg hydrogels was assessed by confocal microscopy. To prepare fluorescently labeled HA, HA–FITC was synthesized by using carbodiimide chemistry. Briefly, HA–FITC was prepared using N-(3-(dimethylamino)­propyl)-N′-ethylcarbodiimide hydrochloride (EDC) chemistry. A solution of fluoresceinamine (FITC, 5 mg in 20 mL of dimethylformamide, DMF) was added to a solution of HA (50 mg) dissolved in 20 mL of water. *N*-hydroxysuccinimide (NHS, 100 mg) was then added, and the pH was adjusted to 4.75 with 0.01 M HCl. Subsequently, EDC (50 mg) was added to initiate the coupling. The reaction mixture was stirred overnight, followed by dialysis against 100 mM NaCl for 3 days and distilled water for 2 days. The purified product was obtained by freeze-drying. Alg-HA-FITC hydrogels were then produced as described above and maintained in culture medium at 37 °C under 5% CO_2_ for 7 days. HA-FITC distribution within the gels was visualized using a confocal microscope (LSM 980, Zeiss).

### 4.3 Formation and Culture of U-87 Spheroids and Their Combination with HA-Loaded Alg Hydrogels

The GBM cell line U-87 MG (GFP+) was acquired from Creative Biogene (UK). Cells were plated and maintained in Dulbecco’s Modified Eagle Medium (DMEM) F-12 (Gibco, ref: 42400028), supplemented with 10% fetal bovine serum (FBS, Gibco, ref: A3160802) and 1% antibiotic/antimycotic (ATB, Gibco, ref: 15240062), and incubated at 37 °C with 5% CO_2_. After reaching approximately 80% of confluence, cells were washed with PBS and detached from the culture flask by incubation with 1% of TrypLE Express (ref: 12605028, Gibco) for 5 min at 37 °C.

U-87 spheroids were generated in an ultralow attachment 96-well plate (Costar, ref: 7007) at a density of 1.5 × 10^5^ cells in 200 μL of culture medium per well (DMEM/F-12 supplemented with 10% of FBS and 1% of ATB). The plate was incubated at 37 °C with 5% CO_2_ for 3 days until the spheroids reached the growing stage. For U-87 spheroid encapsulation experiments, initial Alg droplets (with the addition/absence of HA of different Mw, approx. 110 μL) were prepared and placed into a QGel mold. Using a syringe, spheroids were carefully extracted from the ultralow attachment plate and inserted into the hydrogel droplet. The QGel mold was then sealed, and a Pasteur pipet was used to inject a CaCl_2_ solution into the mold. The system was left undisturbed for 10 min to allow Alg cross-linking and gelation. Finally, culture medium was added, and the whole system was incubated at 37 °C with 5% CO_2_ for 3 days to promote cell expansion before being transferred for further testing.

The cytotoxicity of different culture media in contact with U-87 spheroids was assessed using a lactate dehydrogenase (LDH) assay following the manufacturer’s instructions. U-87 spheroids were generated as described above and cultured for 3 days in three different media (Astrocyte Medium, Pericyte Medium, and EndoGRO Medium). After incubation, 50 μL of conditioned medium was collected from each well containing a spheroid and mixed with 50 μL of the LDH reagent. The mixture was incubated for 30 min at room temperature, after which the reaction was terminated by adding 50 μL of a stop solution. Absorbance was measured at 490 and 680 nm using a microplate reader (Synergy, Bio-Tek). LDH activity was calculated as the difference between absorbance values at 490 and 680 nm.

### 4.4 Preparation of the BBB In Vitro Model

To create a transwell BBB model, we used immortalized human astrocytes (Innoprot, ref: P10251-IM), human brain vascular pericytes (Innoprot, ref: P10363), human brain microvascular endothelial cells (hBMECs, Innoprot, ref: P10361). Astrocytes and pericytes were cultured and used between passages 7 and 10, while HBMECs were used between passages 3 and 6. During subculture, cells were maintained in a humidified atmosphere at 37 °C and 5% CO_2_, using adequate culture media for each cell type: Astrocyte Medium Kit (Sciencecell, ref: P6010) for astrocytes; Pericyte Medium Kit (Sciencecell, ref: P60121) for pericytes; and EndoGRO-MV Complete Culture Medium Kit (Merck Millipore, ref: SCME004) for hBMECs. After reaching approximately 80–90% of confluence, cells were washed with PBS and detached from the culture flask by incubation with 1% of TrypLE Express (Gibco, ref: 12605028) for 5 min at 37 °C, followed by centrifugation of the cell suspension at 300 g for 5 min. To generate the in vitro BBB model, a 12 mm transwell setup was used, where the insets were composed of a polycarbonate bottom membrane presenting a 0.4 μm pore size (Corning, ref: 3401). Before cell culture, the transwell membrane was coated on the basolateral side (i.e., bottom side of the membrane) of the inset with human collagen Type IV (2 μg/cm^2^, Sigma-Aldrich, ref: C5533) and bovine fibronectin (2 μg/cm^2^, Sigma-Aldrich, ref: F1141). This was achieved by inverting the transwell insert and adding 100 μL of collagen/fibronectin solution to the outer surface of the membrane, during 1 h at RT. After incubation, the insets were gently washed 3x with PBS and returned to their original position. Another 100 μL of the coating of the previous coating solution was then added to the apical side (i.e., top side of the membrane) and left to incubate 1 h at RT. Afterward, the membrane was gently washed with PBS, the inset was inverted, and pericytes and astrocytes were seeded onto the outer surface of the membrane, at a density of 5 × 10^4^ cells of each cell type using a mixed cell suspension (1:1). The inverted insets were placed in the incubator for 1 h at 37 °C and 5% CO_2_. After the seeding of the astrocytes and pericytes, the insets were returned to their original orientation, and a mixed culture medium from both cell types was added to both compartments (i.e., inside and outside of the insets) and incubated at 37 °C and 5% CO_2_ for at least 1 h before hBMEC seeding. Finally, the hBMECs (2.5 × 10^5^ cells) were seeded in the apical side of the inset, and the microvascular endothelial cell culture medium was used on the top of the inset. The culture plates were incubated overnight at 37 °C and 5% CO_2_ to allow the complete adhesion and differentiation of the triculture cells that will serve as the in vitro BBB model. After 4 days of the BBB model establishment, three spheroids from the U-87 cell line were encapsulated in the hydrogels, which were prepared under three distinct experimental conditions: Alg hydrogels loaded with 5 kDa of HA at a concentration of 2 mg/mL; Alg hydrogels loaded with 1.5 MDa of HA (2 mg/mL). The encapsulated spheroids were placed on the bottom of the cell culture plates containing the insets with the triculture BBB model and incubated for more 4 days, at 37 °C and 5% CO_2_.

### 4.5 Rheological Characterization of the HA-Loaded Alg Hydrogels

The impact of loading of HA in the Alg hydrogels on their mechanical performance was assessed by using a rheometer (Kinexus Pro+, Malvern). Measurements were performed using a parallel plate geometry (PU8 SR2620 SS) with a 0.8 mm gap, at a controlled temperature of 25 °C, under a frequency sweep over a range between 0.1 and 10 Hz, and at a constant shear strain of 0.1%. Data sets were collected as 10 points per decade.

### 4.6 Time-Lapse Microscopy of Cell Migration

A fluorescence inverted microscope with incubation (Axio Observer, Zeiss) equipped with a temperature/CO_2_ control unit set at 37 °C and 5% CO_2_ was used to observe U-87 cellular growth and assess the cell migration from the spheroid into the hydrogel. Cell migration was monitored using time-lapse microscopy (TLM) by capturing fluorescence images (488 nm) at 30 min intervals (over a total time frame of 24 h), using a 10× objective and the Zen software (Zeiss). The TLM images were analyzed and quantified by using ImageJ software. In each image, the invasion area was manually traced and the invasion edge was outlined. The selected area (highlighted in yellow, Figure S2) was further processed in ImageJ to quantify invasion, allowing for the assessment of the invasion area.

### 4.7 Immunocytochemistry of Alg-HA-GBM

In the case of the *in vitro* BBB model, immunostaining was executed after 8 days of culture. Briefly, the BBB insets were washed with PBS and fixed with methanol (10 min, −20 °C), and blocked with 5% FBS in a 1% BSA solution in DPBS (1 h, RT). Afterward, the membranes were incubated with the primary antibodies: recombinant anti-GFAP antibody [EPR1034Y] (Abcam, ref: ab68428, 1:100); antialpha smooth muscle actin antibody [1A4] (Abcam, ref: ab7817, 1:100); anti-ZO-1 monoclonal antibody [ZO-1–1A12] (ThermoFisher Scientific, ref: 33–9100, 1:100); anti-VE cadherin antibody (Abcam, ref: ab33168, 1:100); anti-claudin 5 antibody [EPR7583] (Abcam, ref: ab131259, 1:100), and CD31/PECAM-1 antibody (Novus Biologicals, ref: DGX0317021, 1:50). After incubation with the primary antibodies, dissolved in blocking solution (overnight, 4 °C), the membranes were washed with DPBS and incubated with secondary antibodies, namely: Alexa Fluor 594 anti-mouse (1:500 in PBS, 1 h, RT) for ZO-1, α-SMA and CD31 and Alexa Fluor 488 anti-rabbit (1:500 in PBS, 1 h, 4̊C) for GFAP, VE-cadherin, and Claudin-5. DAPI (1:1000) was used to stain the nuclei. Immunofluorescence images were collected using a confocal microscope (LSM 980, Zeiss) and an Upright Microscope (DM6 B, Leica).

### 4.8 Protein Expression by Western-Blot

U-87 spheroids (cultured for 3 days on each hydrogel) were collected, washed with cold PBS, and lysed using 200 μL of RIPA buffer (Sigma), supplemented with protease and phosphatase inhibitor cocktails (cOmplete and PhosSTOP from ROCHE, respectively). Samples were alternately vortexed vigorously for 5 min and subjected to ultrasounds while keeping them on ice (until full spheroid disruption). The resulting lysates were transferred to Eppendorf tubes and centrifuged at 18,000*g* for 16 min at 4 °C. Afterward, 180 μL of the supernatant was transferred to a new tube for SDS page, while 6 μL was mixed with 54 μL of water for protein quantification using the Pierce BCA Protein Assay Kit. Bolt LDS sample buffer and reducing agent (ThermoFisher Scientific) were added to the protein lysates, and the samples were denatured at 95 °C for 5 min. Protein lysates (10 μg per lane) were electrophoretically resolved using 4–12% Bis-Tris Protein Gels (Novex) with MES SDS running buffer (Invitrogen, ref: B000202) and transferred to PVDF membranes (Invitrogen, ref: IB24001) using an iBlot 2 System. The membranes were blocked with 4% BSA (w/v) in Tris-buffered saline with Tween (TBS-T, ThermoFisher Scientific). The primary antibodies recombinant anti-CD44 antibody (Abcam, ref: ab189524, 1:250) or cortactin recombinant rabbit monoclonal antibody (Invitrogen, SC61–08, MA5–32250, 1:1000) were incubated overnight at 4 °C, followed by IRDye 800CW anti-rabbit (1:10000) for 1 h. All of the WB lanes were detected using an Odyssey Fc Imaging System (LI-COR). The intensity of the bands in the different lanes was quantified by using Imagej software. For the cell lysates, bands were quantified by densitometry and normalized to the housekeeping protein GAPDH.

### 4.9 Transendothelial Electrical Resistance (TEER) Measurement

An EndOhm chamber connected to an Epithelial Voltohmmeter (EVOM) was used to take TEER measurements, which are more reproducible than the chopsticks electrode. In this method, TEER readings were obtained every day using an EndOhm-12G chamber (World Precision Instrument, EVM-EL-03–01–02) and an EVOM Manual resistance reader (World Precision Instrument, EVM-MT-03–01), according to the supplier recommendations. After each measurement, the culture medium in both the apical (top of the inset) and basolateral (bottom of the inset) compartments was replaced. The TEER values were calculated using eq 1, where TEER (Ω) represents the electrical resistance measured across the in vitro BBB model, TEER background (Ω) is the resistance measured across the inset with only the coating and in the absence of cells, and *A* is the surface area of the inset, which was set to 1.12 cm^2^:^27^

1 $$TEER\;\left(\Omega \cdot \mathrm{cm}\right)=\left|TEER\;\left(\Omega \right)-TEER\;background\;\left(\Omega \right)\times A\;\left({cm}^{2}\right)\right|$$

### 4.10 Dextran Permeability Assay

The 4 kDa FITC-dextran (Sigma) was prepared at a concentration of 200 μg/mL in endothelial cell culture medium, and 300 μL of this solution was added into the apical chamber (top side of the inset) of the transwell system. After 4 h, samples were collected from the apical chamber, and the fluorescence intensity was recorded using a microplate reader (SYNERGY HT, Bio-Tek, excitation wavelength of 493 nm, and emission wavelength of 517 nm) to assess the extent of permeation. The permeability of the BBB/BBTB was calculated using eq 2 based on the amount of FITC-dextran that migrated and crossed the BBB/BBTB [19]:

2 $$\%\;D_{c}=\frac{D_{i}-D_{f}}{D_{i}}\times 100$$

where *D*_c_ represents the percentage of dextran that cross the membrane of the *in vitro* BBB model; and *D*_i_ is the dextran added initially (200 μg/mL); *D*_f_ is the dextran that cross the membrane.

### 4.11 Statistical Analysis

Results are presented as the mean and standard deviation of independent experiments for each system under testing. Individual comparisons among the various studied conditions were conducted using Student’s *t* test, whereas differences between groups were assessed using two-way analysis of variance (ANOVA). Statistical analyses were performed using GraphPad Prism version 10. Statistical significance was defined at different levels: *p < 0.1, **p < 0.05, and ***p < 0.01.

## Supplementary Material



## Notes

Additional experimental results, including figures with results that support the discussion (PDF)
