# Supplementary material for: Glioblastoma In Vitro Model That Recapitulates the Influence of the Hyaluronan Molecular Weight in Cancer Cell Motility and Permeability of the Blood-Brain Tumor Barrier
Source: ACS Biomater Sci Eng. 2025 Dec 10;12(1):559–66. doi: 10.1021/acsbiomaterials.5c01740 (PMC12818715; doi:10.1021/acsbiomaterials.5c01740)
Supplement: Supplementary file 1 [file ab5c01740_si_001.pdf]

# SUPPLEMENTARY INFORMATION

## Glioblastoma in vitro model that recapitulates the influence of the hyaluronan molecular weight in cancer cell motility and permeability of the blood-brain tumor barrier

Fabiana Andrade,[a,b] Vânia I. B. Castro,[a,b] Sara Amorim,[a,b] Ana R. Araújo,[a,b] , Olga Martinho,[a,b] Natália Alves,[a,b] Rui L. Reis[a,b] and Ricardo A. Pires,[a,b*]

[a] 3B's Research Group, I3Bs – Research Institute on Biomaterials, Biodegradables and Biomimetics, University of Minho, Headquarters of the European Institute of Excellence on Tissue Engineering and Regenerative Medicine, 4805-017 Barco, Portugal

[b] ICVS/3B's–PT Government Associate Laboratory, Braga/Guimarães, Portugal

* E-mail: rpires@i3bs.uminho.pt

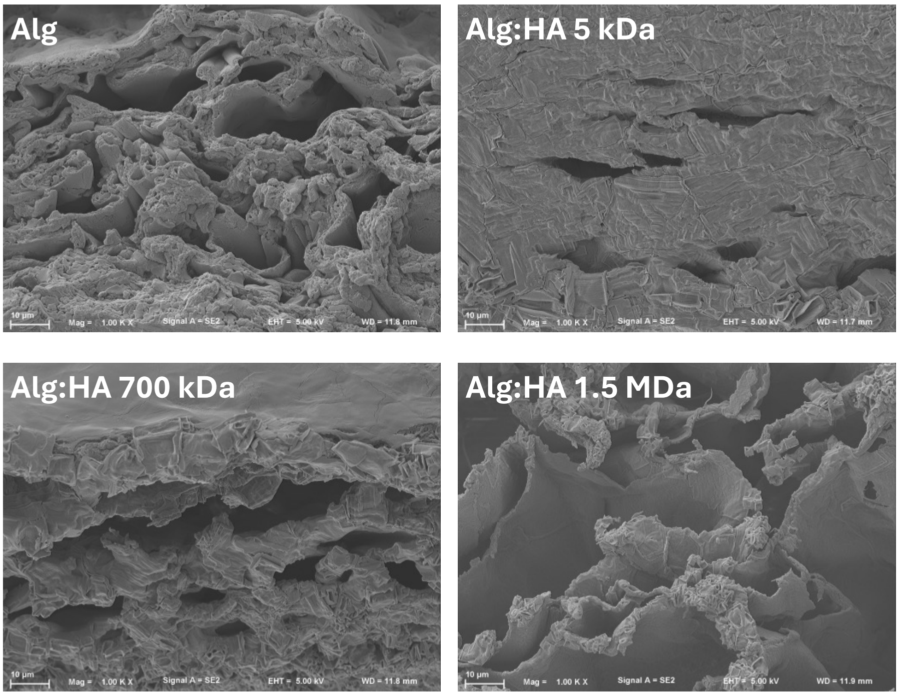


**Figure S1** – SEM images of the Alg hydrogels in the absence/presence of HA of different Mw, i.e., 5kDa, 700kDa and 1.5MDa.

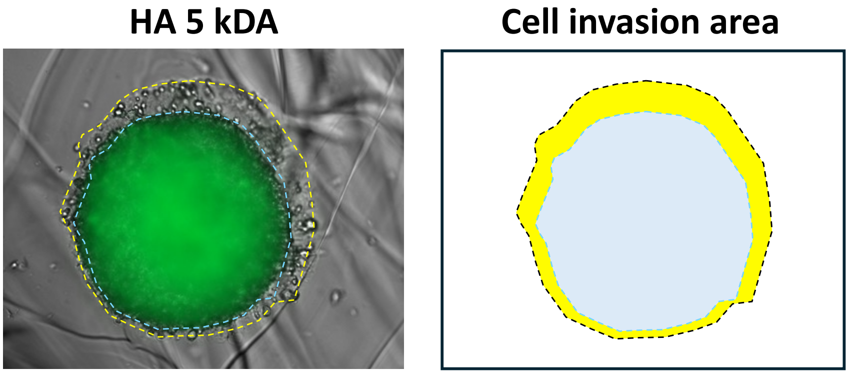


**Figure S2** – Schematic representation of the process used to calculate the cell invasion area in ImageJ. The spheroid boundaries and the invasion edge were outlined; afterwards, the difference between the spheroid and invasion edges (marked in yellow) were quantified by the software.

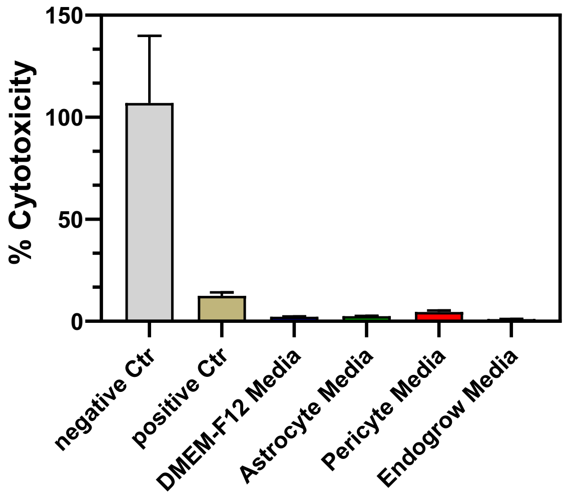


**Figure S3** – Assessment of the impact of the different culture media in the viability of the U87 spheroids at 72h of culture using the LDH quantification method.

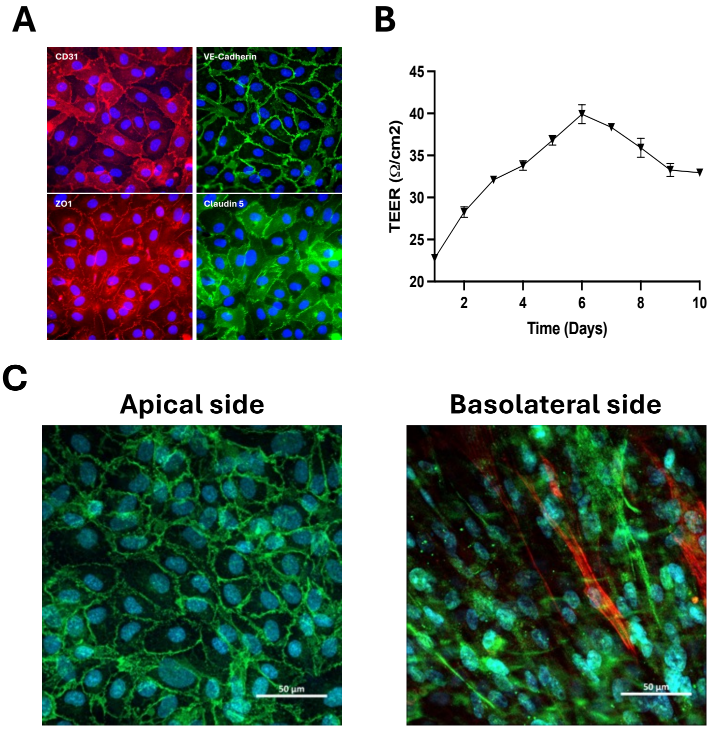


**Figure S4** – **A**. Immunostaining of the hBMECs monolayer (apical side) in the BBB transwell model, showing the expression of CD31, ZO1, VE-Cadherin and Claudin 5. **B**. Transendothelial electrical resistance (TEER) of the BBB model generated by the co-culture of hBMECs, astrocytes and pericytes over 10 days. **C**. Fluorescence images of the apical side of the BBB model, showing the monolayer of hBMECs (stained for VE-Cadherin - green and DAPI/nuclei - blue) and of the basolateral side showing the co-culture of astrocytes (stained for GFAP – green; DAPI/nuclei - blue) and pericytes (stained for α-SMA – red; DAPI/nuclei - blue).

**Alg:HA-FITC 5KDa** **Alg:HA-FITC 1.5MDa**

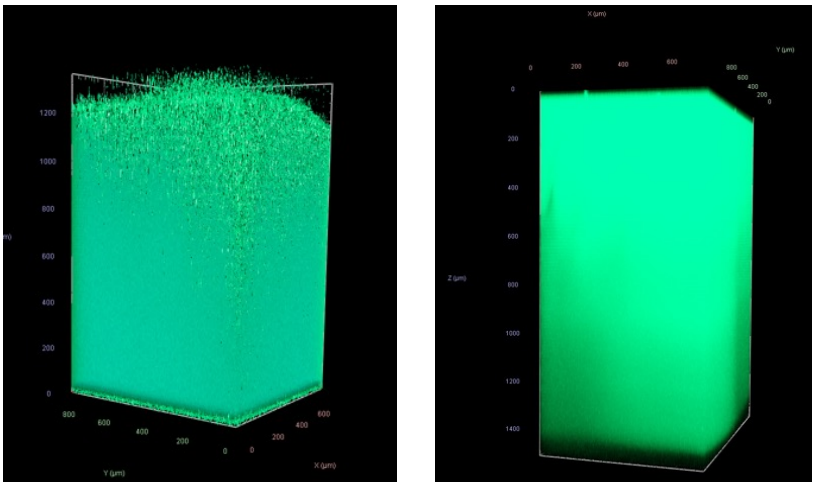

**Figure S5** – Distribution of HA-FITC of 5kDa and 1.5MDa in the Alg hydrogels after 7 days under cell culture conditions.
